# Supplementary figures and images for: Health-related quality of life in a large cohort of patients with cardiac implantable electronic devices A registry-based study
Source: PLoS One. 2024 Dec 23;19(12):e0314978. doi: 10.1371/journal.pone.0314978 (PMC11666060; doi:10.1371/journal.pone.0314978)

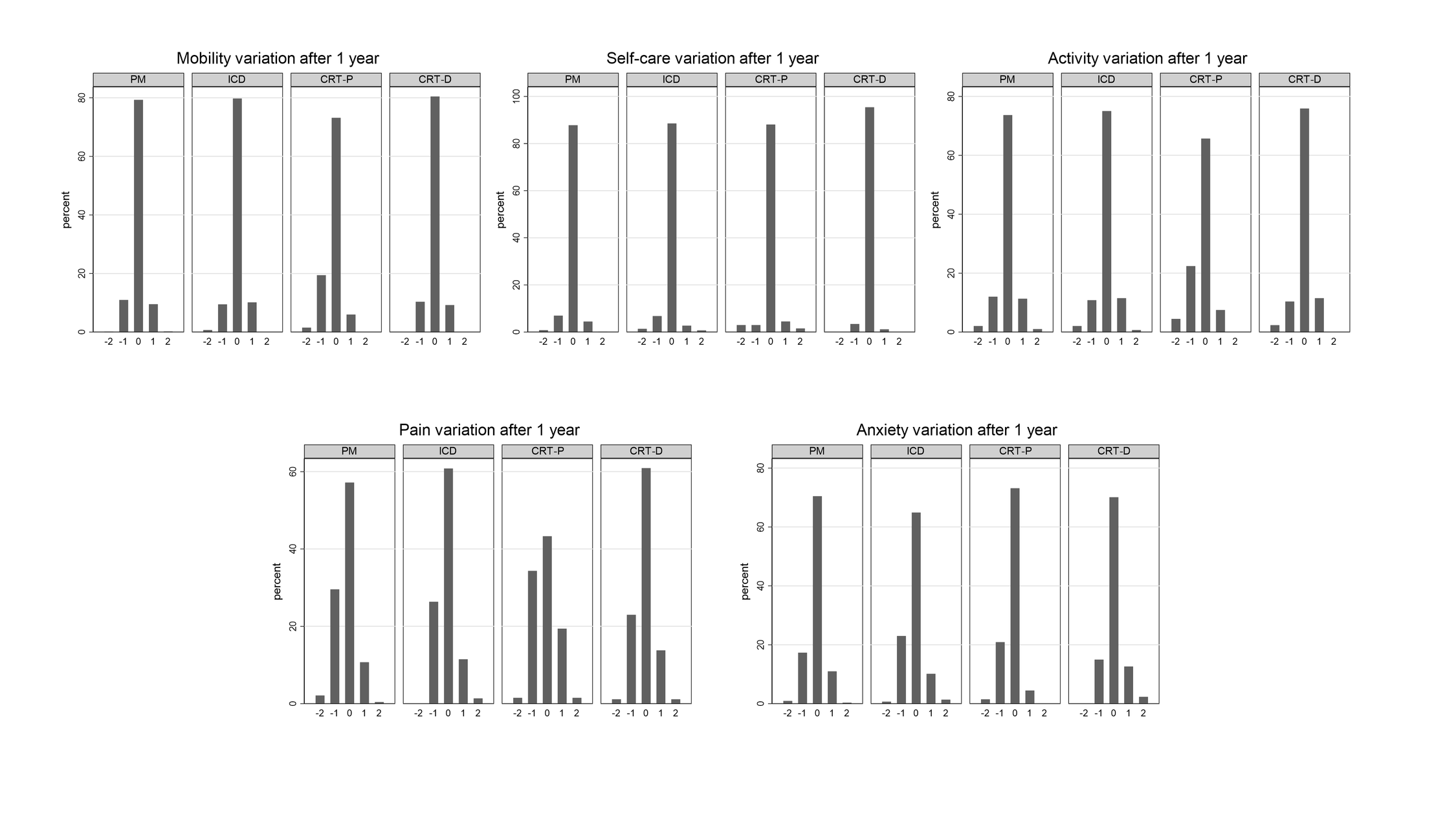

Supplement: S1 Fig — CRT-D = Cardiac resynchronization therapy with defibrillator function, CRT-P = Cardiac resynchronization therapy without defibrillator function, ICD = implantable cardioverter defibrillator, PM = pacemaker. (TIF) [file pone.0314978.s001.tif]

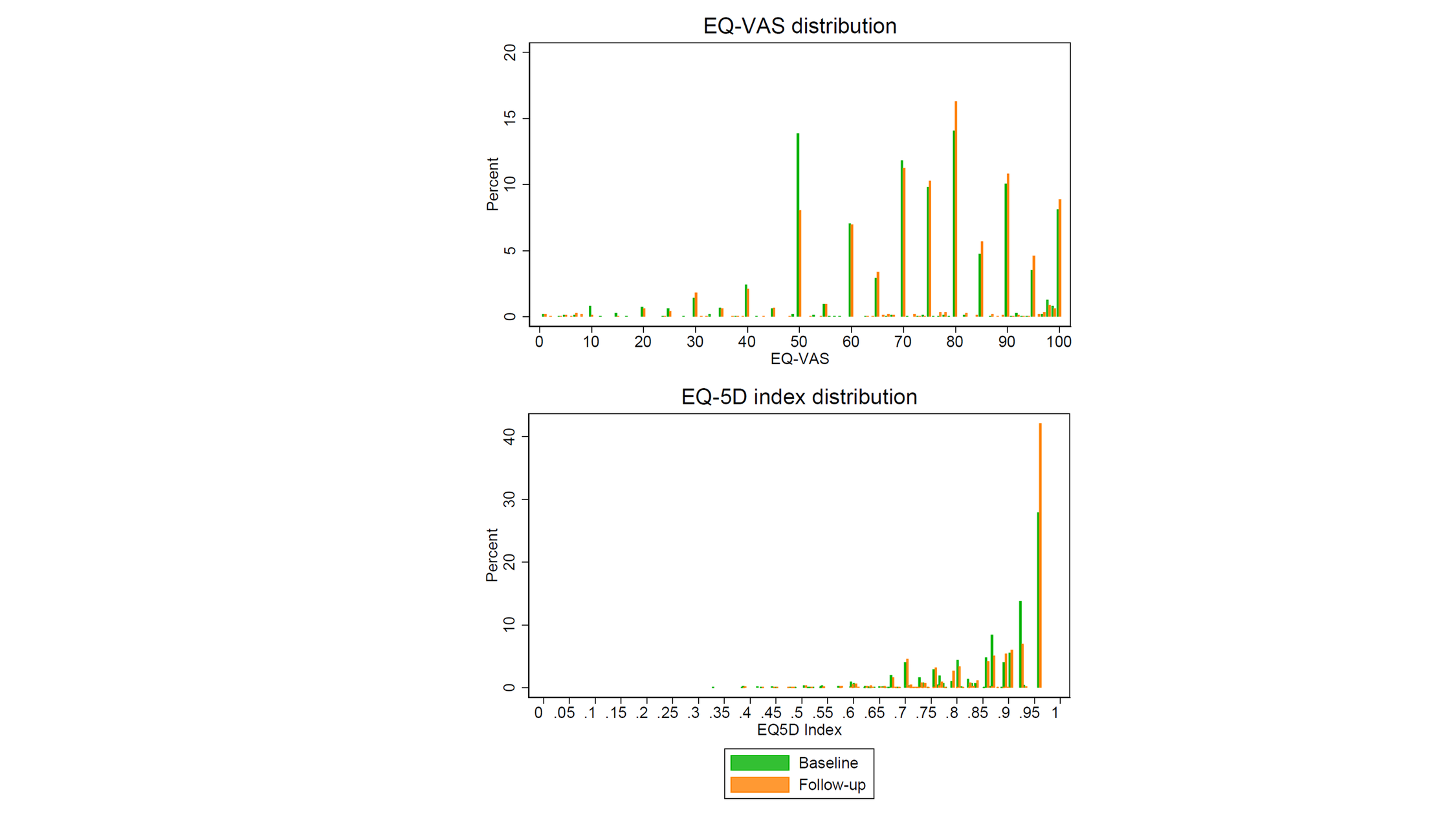

Supplement: S2 Fig — EQ-5D = EuroQol 5-Dimension, EQ-VAS = EuroQol visual analogue scale. (TIF) [file pone.0314978.s002.tif]

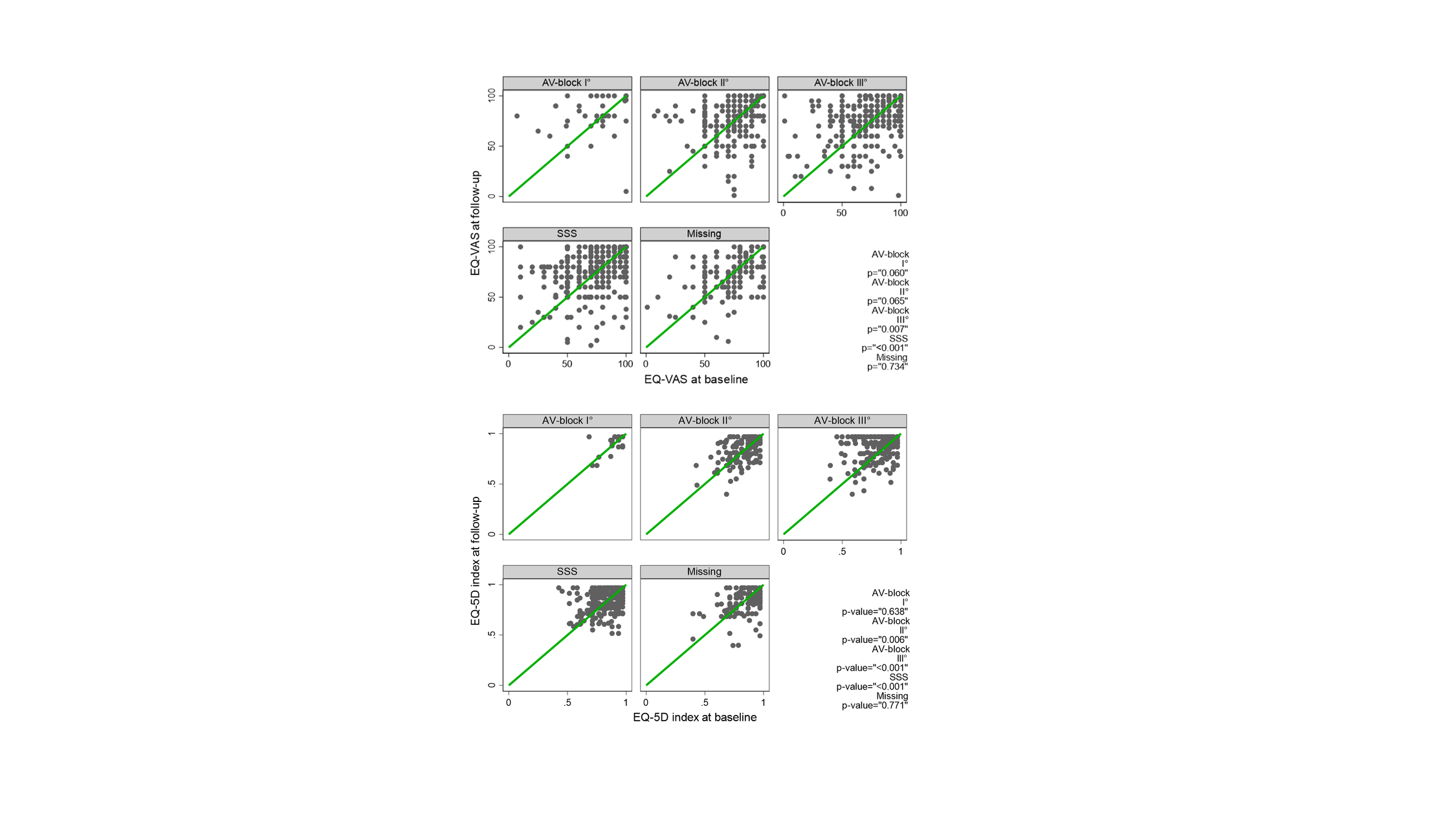

Supplement: S3 Fig — AV = atrioventricular, EQ-5D index = EuroQol 5-Dimension index, EQ-VAS = EuroQol visual analogue scale, SSS = sick sinus syndrome. (TIF) [file pone.0314978.s003.tif]

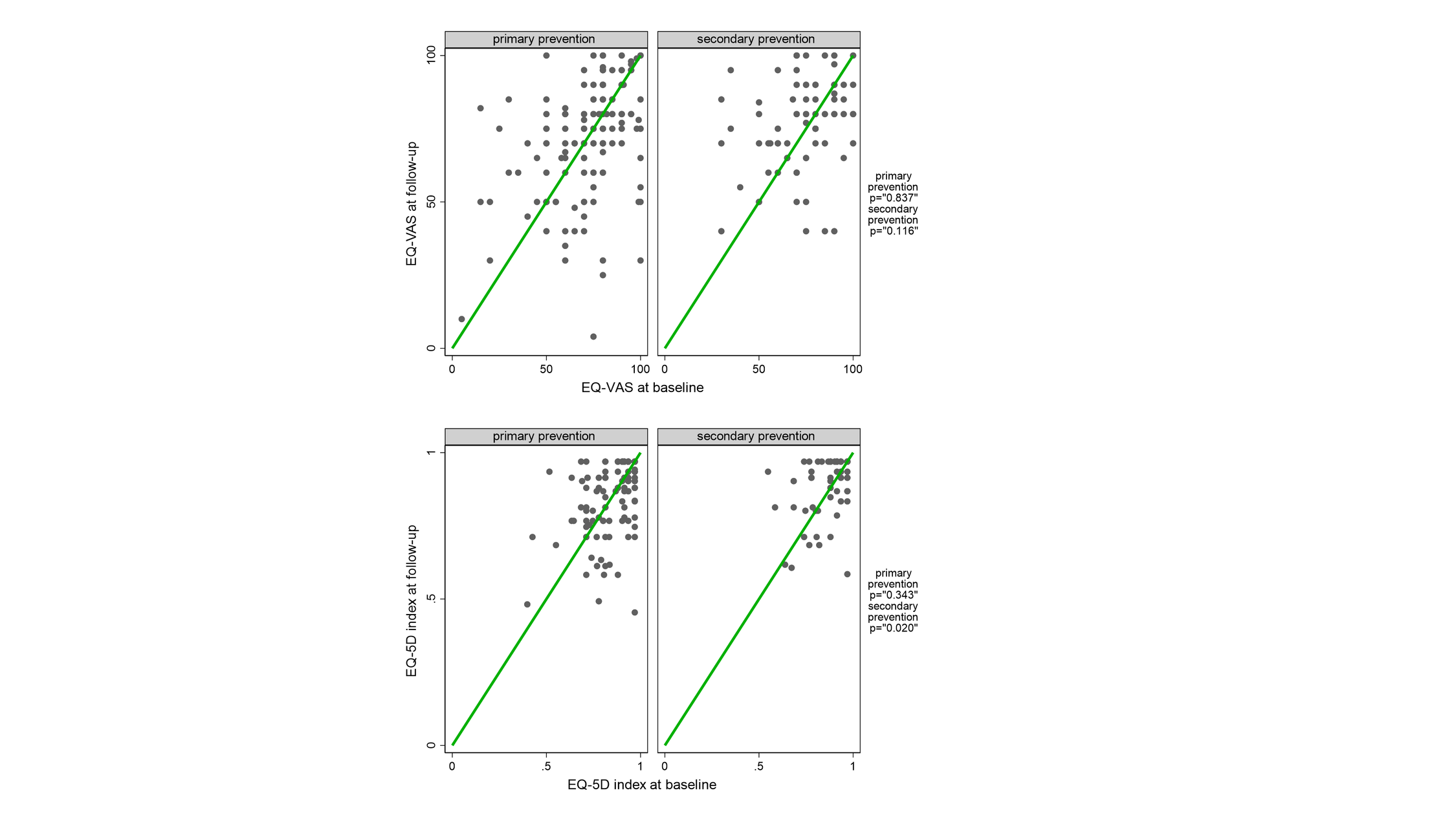

Supplement: S4 Fig — EQ-5D index = EuroQol 5-Dimension index, EQ-VAS = EuroQol visual analogue scale. (TIF) [file pone.0314978.s004.tif]
