# Supplementary material for: Health-related quality of life in a large cohort of patients with cardiac implantable electronic devices A registry-based study
Source: PLoS One. 2024 Dec 23;19(12):e0314978. doi: 10.1371/journal.pone.0314978 (PMC11666060; doi:10.1371/journal.pone.0314978)
Supplement: S1 Table — n = number, CRTD = Cardiac resynchronization therapy with defibrillator function, CRT-P = Cardiac resynchronization therapy without defibrillator function, ICD = implantable cardioverter defibrillator, PM = pacemacker. (DOCX) [file pone.0314978.s005.docx]

**Table S1 EQ-5D dimensions result by device type at baseline and after 1 year**

|  | | | **Pacemaker** | | | | **ICD** | | | | **CRT-P** | | | | **CRT-D** | | | |
| --- | --- | --- | --- | --- | --- | --- | --- | --- | --- | --- | --- | --- | --- | --- | --- | --- | --- | --- |
|  |  |  | **Follow-up** | | | | | | | | | | | | | | | |
|  |  |  | **1** | **2** | **3** | **Total** | **1** | **2** | **3** | **Total** | **1** | **2** | **3** | **Total** | **1** | **2** | **3** | **Total** |
| **Mobility** | **Baseline** | **1** | 621 | 109 | 2 | **732** | 96 | 15 |  | **111** | 31 | 4 |  | **35** | 54 | 8 |  | **62** |
|  |  | **2** | 125 | 309 | 3 | **437** | 13 | 22 |  | **35** | 12 | 17 |  | **29** | 9 | 16 |  | **25** |
|  |  | **3** | 1 | 4 | 3 | **8** | 1 | 1 |  | **2** | 1 | 1 | 1 | **3** |  |  |  | **0** |
|  |  | **Total** | **747** | **422** | **8** | **1177** | **110** | **38** | **0** | **148** | **44** | **22** | **1** | **67** | **63** | **24** | **0** | **87** |
| **Self-care** |  | **1** | 989 | 39 | 1 | **1029** | 129 | 3 | 1 | **133** | 56 | 3 | 1 | **60** | 83 | 1 |  | **84** |
|  |  | **2** | 76 | 37 | 13 | **126** | 9 | 2 | 1 | **12** | 2 | 3 |  | **5** | 2 |  |  | **2** |
|  |  | **3** | 9 | 6 | 7 | **22** | 2 | 1 |  | **3** | 2 |  |  | **2** |  | 1 |  | **1** |
|  |  | **Total** | **1074** | **82** | **21** | **1177** | **140** | **6** | **2** | **148** | **60** | **6** | **1** | **67** | **85** | **2** | **0** | **87** |
| **Activity** |  | **1** | 757 | 113 | 12 | **882** | 95 | 17 | 1 | **113** | 38 | 5 |  | **43** | 56 | 9 |  | **65** |
|  |  | **2** | 120 | 103 | 20 | **243** | 13 | 13 |  | **26** | 13 | 6 |  | **19** | 8 | 10 | 1 | **19** |
|  |  | **3** | 24 | 21 | 7 | **52** | 3 | 3 | 3 | **9** | 3 | 2 |  | **5** | 2 | 1 |  | **3** |
|  |  | **Total** | **901** | **237** | **39** | **1177** | **111** | **33** | **4** | **148** | **54** | **13** | **0** | **67** | **66** | **20** | **1** | **87** |
| **Pain** |  | **1** | 385 | 105 | 5 | **495** | 62 | 17 | 2 | **81** | 15 | 11 | 1 | **27** | 36 | 9 | 1 | **46** |
|  |  | **2** | 322 | 282 | 21 | **625** | 34 | 28 |  | **62** | 19 | 14 | 2 | **35** | 19 | 15 | 3 | **37** |
|  |  | **3** | 25 | 26 | 6 | **57** |  | 5 |  | **5** | 1 | 4 |  | **5** | 1 | 1 | 2 | **4** |
|  |  | **Total** | **732** | **413** | **32** | **1177** | **96** | **50** | **2** | **148** | **35** | **29** | **3** | **67** | **56** | **25** | **6** | **87** |
| **Anxiety** |  | **1** | 630 | 119 | 4 | **754** | 68 | 14 | 2 | **84** | 37 | 3 |  | **39** | 45 | 9 | 2 | **56** |
|  |  | **2** | 187 | 195 | 10 | **392** | 30 | 27 | 1 | **58** | 13 | 12 |  | **25** | 13 | 16 | 2 | **31** |
|  |  | **3** | 11 | 17 | 4 | **32** | 1 | 4 | 1 | **6** | 1 | 1 |  | **2** |  |  |  | **0** |
|  |  | **Total** | **829** | **331** | **18** | **1177** | **99** | **45** | **4** | **148** | **50** | **16** | **0** | **67** | **58** | **25** | **4** | **87** |

**n = number, CRTD = Cardiac resynchronization therapy with defibrillator function, CRT-P = Cardiac resynchronization therapy without defibrillator function, ICD = implantable cardioverter defibrillator, PM = pacemacker**
