# Supplementary material for: Health-related quality of life in a large cohort of patients with cardiac implantable electronic devices A registry-based study
Source: PLoS One. 2024 Dec 23;19(12):e0314978. doi: 10.1371/journal.pone.0314978 (PMC11666060; doi:10.1371/journal.pone.0314978)
Supplement: Attachment — Submitted filename: Rebuttal.docx [file pone.0314978.s006.docx]

Stockholm, August 1st 2024

**To**

**Marcelo Arruda Nakazone, M.D., Ph.D.**

Academic Editor

PLOS ONE

Dear Editor,

Please find enclosed the newly revised version of the manuscript entitled “**Health-related quality of life in in a large cohort of patients with cardiac implantable electronic devices – a registry based study** “[PONE-D-24-18282], which has been resubmitted to your journal and that we hope you will consider for publication.

We are extremely grateful for the efforts of the reviewer on our resubmission, which have permitted a considerable improvement of the manuscript. A point-to-point response to each issue raised is reported below and changes in the “Revised Manuscript with Track Changes” are identified by red font. At this time we confirm that the manuscript is an original work and has neither been published nor is currently under consideration for publication by any other journal. None of the paper’s contents have been previously published by any other journal.

Looking forward to a kind reply at your earliest convenience.

With our personal best regards,

On behalf of all authors,

**Journal requirements:**

1. Please ensure that your manuscript meets PLOS ONE's style requirements, including those for file naming. The PLOS ONE style templates can be found at
   <https://journals.plos.org/plosone/s/file?id=wjVg/PLOSOne_formatting_sample_main_body.pdf> and
   <https://journals.plos.org/plosone/s/file?id=ba62/PLOSOne_formatting_sample_title_authors_affiliations.pdf>

**REPLY**

We adapted the manuscript according to the PLOS ONE’s style requirements.

2. We note that you have indicated that there are restrictions to data sharing for this study. For studies involving human research participant data or other sensitive data, we encourage authors to share de-identified or anonymized data. However, when data cannot be publicly shared for ethical reasons, we allow authors to make their data sets available upon request. For information on unacceptable data access restrictions, please see http://journals.plos.org/plosone/s/data-availability#loc-unacceptable-data-access-restrictions.

**REPLY**

We added the information in the Data Availability Statement on how to obtain data set upon request.

See Availability of data and materials, page 14 line 240

- Data are provided with ethical restriction by the national ICD and pacemaker registry and available on request upon submission of ethical approval. Contact information for data requests is publicly available at <https://www.pacemakerregistret.se/icdpmr/start.do>.

**Reviewer 1:**

Thank you for the opportunity to revise this manuscript. The authors evaluated the association of cardiac implantable electronic devices with health-related quality of life (HRQoL). While the article is grammatically sound, it requires a more comprehensive explanation of the research methodology.

MAJOR COMMENTS:

1. Please describe the statistical analyses performed in the methods section.

**REPLY**

We thank the reviewer for his/her comments. We reported the statistical methods in the “Methods and results” section of the abstract.

See Methods and results, page 2 line 7.

- Data from the Swedish Pacemaker and ICD Registry collected from January 2019 to February 2022 was used to analyze the responses to the European Quality of Life-5 Dimension questionnaire (EQ-5D) before and after one year of CIED implant. Descriptive analysis was performed using Pearson’s chi-square test, the analysis of variance ANOVA, the Kruskal-Wallis test and Wilcoxon signed-rank test when appropriate. A multivariable regression analysis was used to compare EQ-5D index and EQ-VAS variation after 1 year.

2. What is the reference group in these analyses?

**REPLY**

We thank the reviewer for his/her comments. In the descritptive analysis the reference is the baseline when comparing quality of life questionnaire responses before and after the device implante. When comparing different EQ-5D index and EQ-VAS variation by type of device the reference are patient implanted with a PM. We adjusted the abstract as follows to clarify the reference group.

See Methods and results, page 2 line 14

- The EQ-VAS and the EQ-5D index significantly increased after one year from the PM and CRT-P implant (EQ-VAS +2.8, standard deviations (SD) 23 and +5.8, SD 24.9; EQ-5D index +0.019, SD 0.114 and +0.051, SD 0.125) while only the EQ-5D index increased after one year from the ICD implant (+0.002, SD 0.104 ). After adjusting for age, sex and HRQoL at baseline, the presence of defibrillator was associated with lower EQ-VAS (ICD EQ-VAS variation: -3.4, 95% confidence intervals (CI) -6.7; -0.1 and CRT-D EQ-VAS variation -4.8, 95% CI -8.8;-0.7) and EQ-5D index (ICD EQ-5D index variation: -0.018, 95% CI -0.035; -0.0003 and CRT-D EQ-5D index variation -0.025 95% CI 0.046;0.004) after one year compared to PM.

3. In this sentence in lines 14-15 "the presence of defibrillator was associated with lower EQ-VAS (-3.4, 95% confidence intervals (CI) -6.7; -0.1 and -4.8, 95% CI -8.8;-0.7)", what do these coefficients represent?

**REPLY**

We thank the reviewer for his/her comments. The coefficients represent the points of variation in EQ-VAS and EQ-5D index respectively after one year from the implant of ICD and CRT-D.

See Methods and results, page 2 line 17

- After adjusting for age, sex and HRQoL at baseline, the presence of defibrillator was associated with lower EQ-VAS (ICD EQ-VAS variation: -3.4, 95% confidence intervals (CI) -6.7; -0.1 and CRT-D EQ-VAS variation -4.8, 95% CI -8.8;-0.7) and EQ-5D index (ICD EQ-5D index variation: -0.018, 95% CI -0.035; -0.0003 and CRT-D EQ-5D index variation -0.025 95% CI 0.046;0.004) after one year compared to PM.

4. What is the study design?

**REPLY**

We thank the reviewer for his/her comments. The design of our study is a retrospective paired cohort study.

5. Please indicate that HRQoL measurements were conducted twice.

**REPLY**

We thank the reviewer for his/her comments. We change the abstract as follow to specify that measurements were conducted twice.

See Methods and results, page 2 line 7

- Data from the Swedish Pacemaker and ICD Registry collected from January 2019 to February 2022 was used to analyze the responses to the European Quality of Life-5 Dimension questionnaire (EQ-5D) before and after one year of the CIED implant.

6. The authors ought to incorporate a literature review detailing what is already known about the topic, identifying the knowledge gaps in existing literature, and explaining how this study addresses those gaps.

**REPLY**

We thank the reviewer for his/her comments. We addressed the available evidence in the literature and reported the possible contribution to the topic of our study in the introduction session.

See Introduction, page 3 line 36

- Regarding cardiac implantable electronic device (CIED) there are few studies evaluating changes in HRQoL before and after the device implantation. The majority of studies evaluating HRQoL in patient implanted with a PM are based on the effect of different pacing modality. (2–6). Data on ICD and quality of life are more robust and did not show a relevant impact on HRQoL (7–10). CRT devices instead had QoL improvement as a key outcome in randomized controlled trials as MIRACLE, REVERSE and lastly the BUDAPEST trial (11–13).

See Introduction, page 3 line 48

- The purpose of our registry based cohort study, therefore, is to describe the variation of HRQoL after one year from the CIED implantation helping to better understands and characterize the HRQoL domain status and variation of patients implanted with a CIED in a real-life setting.

7. Please provide a comprehensive description of the independent variables in this analysis

**REPLY**

We thank the reviewer for his/her comments. We described the independent variables in the Methods section.

See Methods, page 4 line 65

- Independent variables available were: age expressed in years, sex, reported bradycardic indication for PM implantation (complete atrioventricular block, second-grade atrioventricular block, other atrioventricular blocks, sick sinus syndrome) and primary or secondary prevention indication for ICD implantation, pacing modality (only atrial pacing AAI, only ventricular pacing mode VVI, bicameral pacing DDD) and reported prevalent symptomatology at the implant (no symptoms, dyspnea/asthenia, palpitations, syncope, dizziness).

8. What type of regression was used? This question is particularly important because the outcome variables are bounded (EQ-VAS ranges from 1-100)

**REPLY**

We thank the reviewer for his/her comments. We specified which regression model was used for the two outcome variables.

Se Methods, page 5 line 89

- A multivariable tobit regression analysis was used to compare EQ-5D index and EQ-VAS variation after 1 year from the implant adjusting for age, sex and HRQoL at baseline.

9. What is the study design?

**REPLY**

We thank the reviewer for his/her comments. The design of our study is a retrospective paired cohort study.

See Methods, page 5 line 82

- In our retrospective paired cohort registry study, categorical variables were presented as absolute numbers (%) and continuous variables as mean [standard deviation (SD)] if normally distributed or medians [interquartile ranges (IQR)] if not normally distributed.

MINOR COMMENTS:

1. Line 4: Please remove the comma after "(CRT-P)" and change" [on] health-related quality of life (HRQoL)" to "[with] health-related quality of life (HRQoL) "

**REPLY**

We thank the reviewer for his/her comments. We changed the text as properly suggested.

Se Abstract introduction, page 2 line 5

- The association of cardiac implantable electronic devices (CIED), namely pacemaker (PM), implantable cardioverter-defibrillator (ICD) and cardiac resynchronization therapy with (CRT-D) or without defibrillator (CRT-P) with health-related quality of life (HRQoL) is lacking.

2. Please replace "February 2022 to analyze" with "February 2022 was used to analyze"

**REPLY**

We thank the reviewer for his/her comments. We changed the text as properly suggested.

Se Abstract introduction, page 2 line 7

- Data from the Swedish Pacemaker and ICD Registry collected from January 2019 to February 2022 was used to analyze the responses to the European Quality of Life-5 Dimension questionnaire (EQ-5D) before and after one year of CIED implant.

3. Please consider rephrasing this sentence to make the meaning of "prevalence of females" clearer. Does it mean the proportion of females? If so, the sentence could be revised as follows: "The median age was 77 years with females constituting 38% of the PM group and 17% of the ICD group.

**REPLY**

We thank the reviewer for his/her comments. We changed the text as properly suggested.

Se Abstract introduction, page 2 line 13

- The median age was 77 years with females constituting 38% of the PM group and 17% of the ICD group.

**Reviewer 2:**

This study asks and addresses an important question – what are the effects of cardiac implantable electronic devices (CIED) on the well-being of patients? Importantly, this assessment of well-being is performed by the patients themselves. This paper is well written and presents a series of well-explained and sensible analyses. Understanding the impact of various variables on post-implantation well-being is clearly important, and this paper makes an important contribution to this area of inquiry.

1. It is interesting (and surprising to me) that the baseline HRQoL values seem so high? For example, the median baseline EQ-5D index is greater than 0.9. Isn’t this a surprisingly high number for patients about to undergo surgery? I think it would be useful for the authors to discuss this and comment on the range of values observed in other relevant groups e.g. healthy population.

**REPLY**

We thank the reviewer for his/her comments. We agree that discuss the results of baseline HRQoL and its comparison with other population is fundamental and improve the overall quality of our manuscript and we add a paragraph on the discussion accordingly.

Se Discussion, page 10 line 154

- The first interesting finding of our study was the high EQ-5D index and EQ-VAS value at baseline. Our cohort of patients mainly implanted with pacemaker had an HRQoL at baseline comparable with the general Swedish population (25). In the study of Inácio N.A. et al. on elderly patients with pacemakers the median EQ5D index was lower but similar EQ-VAS was reported (26). Nevertheless, a direct comparison with other studies on CIED is difficult due to the variety of population groups and questionnaires utilised (SF36, MLHF, MacNew scale) (27–29). Other than patient characteristics and comorbidities that have a heavy impact on HRQoL, environmental factors may play a role. We think that the online subministration of the questionnaire allowing the completion at home, in a less stressful environment may have also impacted our results.

MINOR COMMENTS:

1. Typo in title! “in in”

**Reply**

We thank the reviewer for his/her note, we corrected the typo.

2. Change “one year the” to “one year of the”

**Reply**

We thank the reviewer for his/her comment, we changed as suggested
