# Supplementary material for: Health-related quality of life in a large cohort of patients with cardiac implantable electronic devices A registry-based study
Source: PLoS One. 2024 Dec 23;19(12):e0314978. doi: 10.1371/journal.pone.0314978 (PMC11666060; doi:10.1371/journal.pone.0314978)
Supplement: Attachment — Submitted filename: Rebuttal (2).docx [file pone.0314978.s007.docx]

Stockholm, August 1st 2024

**To**

**Marcelo Arruda Nakazone, M.D., Ph.D.**

Academic Editor

PLOS ONE

Dear Editor,

Please find enclosed the newly revised version of the manuscript entitled “**Health-related quality of life in in a large cohort of patients with cardiac implantable electronic devices – a registry based study** “[PONE-D-24-18282], which has been resubmitted to your journal and that we hope you will consider for publication.

We are extremely grateful for the efforts of the reviewer on our resubmission, which have permitted a considerable improvement of the manuscript. A point-to-point response to each issue raised is reported below and changes in the “Revised Manuscript with Track Changes” are identified by red font. At this time we confirm that the manuscript is an original work and has neither been published nor is currently under consideration for publication by any other journal. None of the paper’s contents have been previously published by any other journal.

Looking forward to a kind reply at your earliest convenience.

With our personal best regards,

On behalf of all authors,

**Review Comments:**

**Reviewer 3:**

1. “Since its preventive nature instead of physical symptomatology treatment, may no impact patients' reported outcomes… Furthermore, the fear of inappropriate shocks, a higher
   rate of complication and restrictions on driving license and specific activities may have
   counteracted other beneficial effects perceived by the patients”
   Very confusing statement to me since I see only grouped analysis of ICD implants without break down for primary and secondary prevention and I think these should that be analyzed separately

**REPLY**

We thank the reviewer for his/her comments. We agree with the reviewer that the sentence as written was unclear; the term "preventive nature" is misleading. The intent was to emphasize the different nature of the defibrillation function compared to pacing. For this reason, we have modified the paragraph accordingly

See Discussion, page 10 line 165.

- The defibrillation function by its nature differs from pacing, as it is comparable to insurance. While it is adopted based on the patient's risk of sudden death, the hope is that it will not need to be used**.** For this reason, the lack of tangible effects on symptoms in the absence of a precipitating event that triggers its activation, along with **the fear of inappropriate shocks, a higher rate of complication and restrictions on driving license and specific activities may have counteracted other beneficial effects perceived by the patients.**

2. “Our study showed a significant improvement in HRQoL that was not replicated in the
   primary prevention indication. These findings may show, as reported in the European Heart
   Rhythm Association survey on long-term HRQoL and acceptance of implantable ICD (32), that
   patients who experience major arrhythmias had a larger psychological benefit from the security provided by the defibrillator who may overcome the fear of complication”

   Again, I do not see the comparison. What worries me is that secondary prevention was an indication in nearly half of the ICD group

**REPLY**

We thank the reviewer for his/her comments. Indeed, as suggested by the reviewer, a comparison of subgroups, such as between primary and secondary prevention, especially in an observational study does not allow for conclusions. Despite this, we considered it useful to present our results in the supplementary material with the intent of describing the population and generating hypotheses.

As rightly pointed out, the percentage of patients with ICDs implanted for secondary prevention is high compared to other countries. For this reason, we have reduced the emphasis on subgroup results in our discussion and highlighted the limitations in the limitations section.

See Discussion, page 11 line 178, 189, 195

- **Despite the limitation of subgroup analysis, we described available subgroups in our population such as different symptoms and indications at the CIED implant, to generate hypotheses and further advance the knowledge of patients' reported outcomes.**

**… This finding may suggest the possibility that strong and clear indications may not only have a greater impact on biological outcomes but also on patients’ perspectives.**

**… These findings may raise the hypothesis, as suggested in the European Heart Rhythm Association survey on long-term HRQoL and acceptance of implantable ICD, that patients who experience major arrhythmias had a larger psychological benefit from the security provided by the defibrillator who may overcome the fear of complications.**

See Limitation, page 13 line 2020

Patients’ characteristics and device indications such as the percentage of secondary and primary prevention for defibrillators reflect the Swedish health care system and may not apply to other countries.

3. No worsening of symptoms of quality of life needs to be highlighted I consider that a major finding of this study. My view is that any improvement is a bonus for many patients, particularly those with heart failure.

**REPLY**

We thank the reviewer for his/her comments. We agree that no worsening of quality of life in patients receiving a device is the most important result.

Se Coclusion, Page 13 line 232

The picture provided by this large real-world cohort shows no worsening of HRQoL after CIED implant and strengthens the importance of including the HRQoL in the decision to implement a device, and further study designs.
